# Profils de Sensibilité aux Antibactériens des Isolats Sanguins Chez 74 Patients Infectés Par le VIH Hospitalisés à la Clinique des Maladies Infectieuses et Tropicales du Chu de Fann, Dakar de 2013 à 2016

**DOI:** 10.48327/mtsibulletin.n1.2021.88

**Published:** 2021-04-07

**Authors:** B. Shinga Wembulua, A. Lakhe, K. Diallo Mbaye, N. Ndikou Aw, V.M.P. Cisse Diallo, D. Ka, L. Fortes, M. Seydi

**Affiliations:** Service des maladies infectieuses et tropicales, CHU de Fann, BP 5035, Dakar, Sénégal

**Keywords:** Bactériémie, Sensibilité aux médicaments, Phénotype de résistance, Patients infectés par le VIH, Hôpital, Dakar, Sénégal, Afrique subsaharienne, Bacteremia, Drug susceptibility, Resistance phenotype, HIV-infected patients, Hospital, Dakar, Senegal, Sub-Saharan Africa

## Abstract

**Introduction:**

La bactériémie est associée à une létalité élevée chez les patients infectés par le VIH (PVVIH). La prophylaxie au cotrimoxazole et le mésusage des antibiotiques favorisent la résistance antibactérienne. Seules quelques études ont examiné ce problème chez les PVVIH. Ainsi, l'objectif de cette étude était de décrire le profil de résistance des germes responsables des bactériémies dans cette population.

**Matériel et méthodes:**

Il s'agit d'une étude rétrospective transversale et descriptive menée à la clinique des maladies infectieuses et tropicales du CHU de Fann de mars 2013 à décembre 2016. Les données ont été collectées à partir des dossiers des patients grâce à un questionnaire d'enquête préétabli.

**Résultats:**

Soixante-quatorze cas de bactériémie ont été enregistrés, dont 51,4% chez les femmes. L'âge médian était de 45 ans [18-73 ans] et le taux moyen de CD4 de 83,3 cellules/μl. Les staphylocoques à coagulase négative étaient les plus isolés (14%) suivis d'*Escherichia coli* (10%) et de *Klebsiella pneumoniae* (10%). Les taux de résistance à la méthicilline pour les staphylocoques à coagulase négative et *Staphylococcus aureus* étaient respectivement de 35,7% (5/14) et 22% (2/9). Les souches productrices de BLSE étaient représentées par *Escherichia coli* 50% (5/10), *Klebsiella pneumoniae* 40% (4/10) et *Enterobacter* sp 25% (2/8). Les *Pseudomonas* sp étaient les principaux (22,2%) germes résistants aux carbapénèmes.

**Conclusion:**

Les résultats de cette étude prônent la nécessité d'une surveillance continue de la résistance antibactérienne chez les PVVIH et l'antibiothérapie probabiliste s'appuyant sur les données de surveillance.

## Abstract (en)

**Introduction:**

Bacteremia is associated with high lethality in HIV-infected patients. The widespread use of cotrimoxazole prophylaxis and misuse of antibiotics promote antibacterial resistance. Only few studies have considered this issue in HIV-infected patients. Thus, the objective of this study was to describe the etiology and antibacterial susceptibility patterns of bloodstream isolates in patient living with HIV.

**Material and methods:**

This is a retrospective cross-sectional and descriptive study conducted at the clinic of Infectious and Tropical Diseases of Fann university hospital from March 2013 to December 2016. Data were collected from patients' files according to a pre-establish survey form made of demographic, clinical, bacteriological and biological parameters.

**Results:**

Seventy-four cases of bacteremia were registered, 51.4% of which in women. Participants' median age was 45 years old [18-73 years old] and average CD4 count 83.3 cells/μl. The most commonly isolated bacteria were coagulase negative staphylococci (14%) followed by *Escherichia coli* (10%) and *Klebsiella pneumoniae* (10%). Rates of methicillin resistance for coagulase negative staphylococci and *Staphylococcus aureus* were 35.7% (5/14) and 22% (2/9), respectively. The most frequent ESBL producing germs were *Escherichia coli* 50% (5/10), *Klebsiella pneumoniae* 40% (4/10) and *Enterobacter* sp 25% (2/8). *Pseudomonas* sp were the most (22.2%) germs resistant to carbapenems.

**Conclusion:**

The result of this study advocates the need for ongoing surveillance of antibacterial resistance in HIV-infected patients and empirical antibiotic therapy based on surveillance data.

## Introduction

Les personnes vivant avec le VIH (PVVIH) sont disproportionnellement exposées aux infections bactériennes en raison du déficit à la fois de l'immunité humorale, cellulaire et cutanéomuqueuse [20]. Plus de 20% de ces infections consistent en une bactériémie dont le pronostic est souvent réservé [20, 23]. Par ailleurs, les options thérapeutiques sont réduites au fils des années par l'émergence des bactéries résistantes aux antibiotiques usuels. Aux États-Unis, les infections à *Staphylococcus aureus* résistant à la méthicilline (SARM) sont environ 10 fois plus fréquentes chez les PVVIH que dans la population générale [9]. Dans les pays à ressources limitées, la prophylaxie au cotrimoxazole, l'automédication, les erreurs diagnostiques et la pauvreté constituent les principaux facteurs contribuant à ces résistances [3, 13]. Les études analysant le profil de résistance des isolats sanguins, spécifiquement chez les patients infectés par le VIH sont rares car la plupart d'entre elles concernent la population générale [5].

Cette étude, une première dans notre service depuis la revue de Seydi et al il y a dix ans [19], vise à décrire le profil de résistance des germes responsables des bactériémies chez les patients adultes infectés par le VIH.

## Matériel et Méthodes

Il s'agit d'une étude rétrospective transversale et descriptive menée au service des maladies infectieuses et tropicales (SMIT) du CHU de Fann à Dakar, Sénégal de mars 2013 à décembre 2016. Ce dernier est un hôpital de niveau tertiaire fournissant à la fois des services hospitaliers et ambulatoires liés aux maladies infectieuses.

Tous les patients séropositifs adultes (≥ 18 ans) hospitalisés présentant une hémoculture positive ont été inclus dans l'étude. Les données ont été collectées à partir des dossiers des patients à l'aide d'un questionnaire d'enquête préétabli reprenant les paramètres socio-démographiques (âge, sexe), clinico-biologiques (statut VIH et profil, numération CD4) ainsi que bactériologiques (germes isolés, profils de résistance).

### Culture et diagnostic des bactériémies

Des échantillons de sang de 10 ml ont été prélevés chez les PVVIH présentant de la fièvre ou des frissons puis inoculés dans des flacons d'hémoculture. Ces échantillons ont ensuite été envoyés au service de bactériologie de l'hôpital de Fann où la turbidité (signe de croissance bactérienne) était quotidiennement recherchée jusqu'au 5^e^ jour. Les bouillons de culture troubles ont ensuite été inoculés dans des plaques de gélose au sang et des plaques de gélose au chocolat (Bio-Rad laboratories, Richmond, CA), puis incubés à 37 °C pendant 16-24 heures. Les cultures incubées à la gélose au chocolat ont été réalisées dans une atmosphère microaérophile en utilisant un pot à bougie. Le diagnostic de bactériémie était posé en présence d'au moins un flacon d'hémoculture positif. L'identification des bactéries a été faite en se basant sur les caractéristiques des colonies, la réaction Gram des organismes et les tests biochimiques (galeries standards) suivant les procédures standard.

### Test de sensibilité aux antimicrobiens

Un antibiogramme a été réalisé pour tous les isolats d'hémoculture en utilisant la méthode de diffusion sur disque selon les recommandations du comité d'antibiogramme de la Société française de microbiologie (CASFM) [7]. Le profil SARM (*Staphylococcus aureus* résistant à la méthicilline) était attribué aux isolats de *Staphylococcus aureus* non sensibles à l'oxacilline et/ou à la céfoxitine. Celui de BLSE (bactéries sécrétrices des bétalactamases à spectre étendu), aux souches résistantes aux pénicillines, aux céphalosporines et à l'aztréonam, mais sensible à l'amoxicilline - acide clavulanique (ticarcilline - acide clavulanique pour *Pseudomonas* sp et *Enterobacter* sp). La résistance aux carbapénèmes, en cas de résistance à l'imipénem.

### Analyse des données

Les données ont été codées et analysées à l'aide du logiciel Epi-info version 7.2.2.6. Les variables qualitatives ont été exprimées en termes de fréquence et de pourcentage des données saisies et les variables quantitatives sous forme des moyennes ± écart-type ou des médianes et interquartiles selon le type de distribution. Suite aux données manquantes, les taux de résistance (%) présentés dans le tableau III ont été obtenus en rapportant pour chaque antibiotique, le nombre des cas de résistance observés au nombre total de fois où cet antibiotique a été testé.

## Résultats

Durant notre période d'étude, 117 cas d'hémocultures positives ont été enregistrés dont 74 (63,2%) chez les patients infectés par le VIH. Les caractéristiques sociodémographiques des patients, leurs taux de CD4 et la notion de prise d'antirétroviraux (ARV) sont résumés dans le tableau I.

**Tableau I:** Caractéristiques sociodémographiques, taux de CD4 et notion de prise d'ARV chez les PVVIH présentant une bactériémie au SMIT de Fann, Dakar de 2013 à 2016 Socio-demographic characteristics, CD4 range and antiretroviral status of HIV-infected patients with bacteremia at the clinic of infectious and tropical diseases of Fann University hospital, Dakar from 2013 to 2016

Paramètres		Nombre de bactéries (%)
**Sexe**	féminin	38 (51,4)
	masculin	36 (48,6)
**Âge (années)**	18-40	29 (39,2)
	41-59	40 (54,1)
	≥ 60	05 (6,7)
**Taux de CD4 (cell/µl)**	> 200	08 (10,8)
	50 - 200	17 (23,0)
	< 50	41 (55,4)
	NP*	8 (10,8)
Sous ARV**	oui	34 (45,9)
	non	40 (54,1)

* NP: non précisé

** ARV: antirétroviraux

Sur les 74 participants à l'étude, 38 (51,4%) étaient des femmes et 36 (48,6%) étaient des hommes avec un sexe ratio de 1:1,05. L'âge médian était de 45 ans [18-73 ans].

Le taux moyen de CD4 était de 83,3 cellules/μl (intervalle interquartile: 11-107). Plus de la moitié des patients (78,4%) avait un taux de CD4 <200 cellules/μl.

Seuls 34 patients (45,9%) étaient sous traitement antirétroviral (TAR) au moment du recrutement dans l'étude.

### Isolats bactériens et résistance aux antibiotiques

Au total, 100 bactéries ont été isolées. Il s'agissait majoritairement (70%) de bactéries à Gram négatif. Les staphylocoques à coagulase négative (SCoN) (14%), *Escherichia coli* (10%) et *Klebsiella pneumoniae* (10%) étaient les plus isolés. Les tableaux 2 et 3 résument les taux de résistance aux antibiotiques des principaux isolats sanguins ainsi que leurs phénotypes de résistance.

**Tableau II:** Isolats sanguins et phénotypes de résistance aux antibiotiques chez les PVVIH présentant une bactériémie au SMIT de Fann, Dakar, de 2013 à 2016 Antibiotic-resistance phenotypes of bacterial isolates from HIV-infected patients with bacteremia at the clinic of infectious and tropical diseases of Fann University hospital, Dakar, from 2013 to 2016

Paramètres		Nb de bactéries (%)
**Coloration de Gram**	Gram négatif	70 (70)
	Gram positif	30 (30)
**Isolats bactériens**	SCoN*	14(14)
	*Escherichia coli*	10 (10)
	*Klebsiella pneumoniae*	10 (10)
	*Staphylococcus aureus*	9 (9)
	*Pseudomonas* Sp	9 (9)
	*Enterobacter* sp	8 (8)
	*Salmonella* sp	4 (4)
	Groupe D *Streptococcus*	4 (4)
	Streptocoque non groupable	3 (4)
	*Flavobacterium* sp	3 (3)
	*Acinobacter* sp	2 (2)
	*Serratia* sp	1 (1)
**Phénotypes de résistance**	SCoN résistant à la méthicilline	5 (35,7)
	*S. aureus* résistant à la méthicilline (SARM**)	2 (22,2)
	*Escherichia coli* BLSE*** +	5 (50)
	*Klebsiella pneumoniae* BLSE +	4 (40)
	*Enterobacter* sp BLSE +	2 (25)
	*Acinetobacter* sp résistant aux béta-lactamases	1 (50)
	*Klebsiella pneumoniae* résistant aux carbapénèmes	1 (10)
	*Pseudomonas* sp résistant aux carbapénèmes	2 (22,2)
	*Enterobacter* sp résistant aux carbapénèmes	1 (12,5)
	*Escherichia coli* résistant aux carbapénèmes	1 (10)

* SCoN (staphylocoques à coagulase négative): *Staphylococcus saprophyticus* (8), *Staphylococcus epidermidis* (6)

** SARM: *Staphylococcus aureus* résistant à la méthicilline

*** BLSE béta-lactamases à spectre étendu

**Tableau III:** Résistance des principaux isolats sanguins aux autres familles d'antibiotiques chez les PVVIH hospitalisés au service des maladies infectieuses et tropicales du CHU de Fann, Dakar, de 2013 à 2016 Resistance of main blood isolates to other families of antibiotics in HIV-infected patients with bacteremia at the clinic of infectious and tropical diseases of Fann University hospital, Dakar, from 2013 to 2016

Isolats sanguins	CTX	TE	CHL	PEF	CIP	PR	E	GM	AN	VA	AMC	AMX	CAZ	PIP
**Bactéries à Gram+**														
*Staphylococcus* **sp**	8 (66,7)*	7 (50)	3 (21,4)	8 (57,1)	NA	2 (10,5)	7 (36,8)	4 (23,5)	NA	0 (00)	4 (30,7)	NA	NA	NA
*Streptococcus* **sp**	NA	5 (100)	3 (100)	2 (66,7)	NA	4 (100)	4 (100)	3 (75)	NA	0 (00)	NA	1 (20)	NA	NA
**Bactéries à Gram-**														
*Escherichia coli*	3 (100)	1 (100)	1 (16,6)	1 (50)	3 (60)	NA	NA	2 (50)	1 (12,5)	NA	NA	6 (100)	3 (75)	3 (100)
*Klebsiella* **sp**	5 (85,3)	NA	1 (14,3)	NA	2 (33,3)	NA	NA	4 (66,7)	1 (33,3)	NA	0 (100)	6 (100)	5 (100)	4 (100)
*Enterobacter* **sp**	3 (100)	NA	1 (16,7)	NA	1 (16,7)	NA	NA	3 (60)	5 (100)	NA	0 (100)	5 (100)	3 (50)	3 (75)
*Pseudomonas* **sp**	6 (100)	NA	4 (100)	0 (100)	0 (100)	NA	NA	2 (50)	0 (100)	NA	NA	NA	4 (66,6)	3 (75)

Les souches de staphylocoque ont montré une résistance élevée au cotrimoxazole (66,7%), à la péfloxacine (57,1%) et à l'amoxicilline-acide clavulanique (30,7%). La vancomycine reste efficace dans 100% des cas. Les staphylocoques à coagulase négative résistants à la méthicilline représentaient 35,7% (5/14) et les SARM 22,2%. Les souches de streptocoque quant à elles, étaient résistantes à la pristinamycine, la gentamicine (75%) et la péfloxacine (66,7%). Des faibles taux de résistance ont été observés avec la vancomycine et l'amoxicilline (0 et 20%). Pour les souches d'entérobactéries, la résistance était élevée, de l'ordre de 100% pour le cotrimoxazole (*Escherichia coli* et *Enterobacter* sp) et 16 à 60% pour la ciprofloxacine. La résistance à l'amoxicilline-acide clavulanique et à l'amikacine était globalement faible. Par ailleurs, *Escherichia coli* 50% (5/10), *Klebsiella pneumoniae* 40% (4/10) et *Enterobacter* sp 25% (2/8) étaient les principaux germes producteurs de BLSE. Les souches de *Pseudomonas* isolées présentaient une résistance élevée à la pipéracilline (30,8%), à la ceftazidime (66,6%) et à la gentamicine (66,6%) alors que la sensibilité aux quinolones était de 100%. Les *Pseudomonas* sp étaient les principaux germes (22,2%) résistant aux carbapénèmes (Tableau II).

## Discussion

Dans notre étude, 63,2% des cas de bactériémie rapportés concernaient les PVVIH. Ceci n'est pas surprenant vu que notre service est le centre national de référence pour la prise en charge de l'infection à VIH. L'âge médian de 45 ans ainsi que la prédominance féminine notés dans notre étude sont conformes au profil général du VIH/sida en Afrique [24]. Étant plus sexuellement actives, les personnes âgées de 15 à 44 ans courent un risque plus élevé de contracter le VIH qui, à la suite, accroît leur susceptibilité aux infections bactériennes [2]. Il a été observé dans cette étude que la majorité (78,4%) des patients avaient un taux de CD4 <200 cellules/μl de sang avec un taux moyen de 83,3 cellules/μl. Cette observation est en accord avec les connaissances déjà établies de la pathogenèse du VIH. Plus le nombre de CD4 est bas, plus le risque d'infections opportunistes sera élevé. Dans presque toutes les études comparables, plus de 80% des patients étaient au stade sida avec un taux moyen de CD4 compris entre 40 et 80 cellules/μl [1, 14, 19]. Le faible taux de CD4 dans notre étude peut s'expliquer par le fait que la plupart (54,1%) de nos patients n'étaient pas encore sous TAR au moment du recrutement.

Les bactéries Gram négatif étaient prédominantes dans notre série. Ceci est en ligne avec d'autres études précédemment menées dans notre service [19] et ailleurs dans le monde [1, 12].

Le profil des isolats d'hémocultures chez les PVVIH est très hétérogène. Ceci varie selon le type d'étude, la situation géographique et l'écologie bactérienne locale. Une étude rétrospective précédemment menée dans notre service sur les étiologies des bactériémies chez les PVVIH sur une durée de onze ans avait rapporté une predominance de *Salmonella* sp (n= 3), *Escherichia coli* (n = 13), *Streptococcus pneumoniae* (n = 9) et *Staphylococcus aureus* (n = 8) [19]. Dans la série de Marwa KJ et al [13], *Escherichia coli* et *Klebsiella* sp étaient les bactéries les plus courantes parmi les bactéries à Gram négatif et *S. aureus* la principale bactérie à Gram positif. Les staphylocoques à coagulase négative (SCoN), *Escherichia coli, Staphylococcus aureus* et *Pseudomonas* sp étaient les plus isolés dans notre étude.

Les (SCoN) sont de plus en plus rapportés comme principales causes des bactériémies, en particulier chez les patients infectés par le VIH [1, 8, 12]. Appartenant pour la plupart à la flore normale de la peau, leur responsabilité clinique reste peu claire. S'agit-il d'une notion d'opportunisme, vu l'immunodépression qui caractérise l'infection à VIH? Est-ce des cas de contamination? Des études plus poussées sont donc nécessaires afin d'apporter plus de lumière sur ces questions. En outre, le plaidoyer pour le strict respect des mesures d'asepsie devrait être sans appel.

Cette étude donne également un aperçu sur les phénotypes de résistance des bactéries isolées dans notre service. Les bactériémies liées au SARM sont dix fois plus fréquentes chez les PVVIH que dans la population générale [9]. Dans notre étude, la prévalence des SARM était de 22,2% (2/9). Ce constat était conforme aux résultats d'autres études menées aux États-Unis d'Amérique dans lesquelles 15 à 22% des souches de *S. aureus* isolées chez des patients VIH étaient résistantes à la méthicilline [10, 18]. Cependant, des prévalences plus élevées que la nôtre ont été rapportée dans certaines études africaines [11, 25]. La pauvreté peut en être l'un des facteurs promoteurs, car une association négative a été scientifiquement établie entre le revenu national brut (RNB) par habitant et la prévalence des SARM [13].

La production de β-lactamases à spectre étendu (BLSE) est un des principaux mécanismes de résistance des bactéries Gram négatif aux β-lactamines. Elle est associée à une morbidité et une mortalité accrues chez les patients immunodéprimés [15, 16]. Dans notre observation, 5 (50%) souches d'*Escherichia coli*, 4 (40%) de *Klebsiella pneumoniae*, 2 (25%) d'*Enterobacter* sp et une des deux souches d'*Acinetobacter* sp isolées, étaient productrices de BLSE. En phase avec nos observations, Rameshkumar et al ont rapporté que *P. aeruginosa, Acinetobacter baumannii, Escherichia coli* et *K. pneumoniae* était les plus fréquentes des souches sécrétrices de β-lactamases à spectre étendu [16]. La prévalence des souches de *Klebsiella* productrices de BLSE était de 51,6% chez les patients VIH suivis à la Calcutta School of Tropical Medicine [6]. Ce défi croissant peut s'expliquer entre autres par le portage fécal, largement rapporté, des bactéries productrices de BLSE chez les patients infectés par le VIH [22], probablement due à la pression sélective liée au recours à la prophylaxie au cotrimoxazole d'une part et à la forte incidence d'infections bactériennes nécessitant une antibiothérapie chez le PVVIH d'autre part. Étant donné que les carbapénèmes sont devenus les molécules de choix contre les bactéries multirésistantes, des cas de résistance à ces molécules sont de plus en plus rapportés [4, 17]. Dans notre observation, les *Pseudomonas* sp étaient les principaux (22,2%) germes résistants aux carbapénèmes. Une étude américaine a confirmé l'augmentation chez les PVVIH, des cas de résistance des souches de *Pseudomonas aeruginosa* aux carbapénèmes, passant de 5,1% en 2010 à 46,2% en 2016 [21]. Ces résultats, associés aux nôtres, attestent un risque accru d'infection par des souches multirésistantes chez les patients infectés par le VIH.

## Conclusion

Les bactériémies sont courantes chez les patients infectés par le VIH. Elles sont principalement dues aux bactéries Gram-négatives (70%). La responsabilité des staphylocoques à coagulase négative reste inconnue.

Outre la surveillance locale et régionale de la résistance aux antimicrobiens, le respect des protocoles d'hygiène hospitalière et l'antibiothérapie probabiliste s'appuyant sur les données de surveillance, restent des mesures fondamentales.

## Conflits D'intérêts

Les auteurs ne déclarent aucun conflit d'intérêt.
